# To Explant or not to Explant Neural Implants: an Empirical Study into Deliberations of Dutch Research Ethics Committees

**DOI:** 10.1007/s12152-025-09619-z

**Published:** 2025-10-10

**Authors:** Katherine Bassil, Karin Jongsma

**Affiliations:** Department of Bioethics and Health Humanities, Julius Center, University Medical Center Utrecht, Utrecht, The Netherlands

**Keywords:** Explantation, Research ethics, Brain computer interfaces, Neurotechnology

## Abstract

Neural implants such as brain-computer interfaces and spinal cord stimulation offer therapeutic prospects for people with neurological and psychiatric disorders. As neural devices are increasingly tested in clinical research, the decision to explant requires carefully weighing both known and unknown medical and psychological risks, necessitating a thorough evaluation of the benefits and risks of each available option. Research Ethics Committees (RECs) play an important role in assessing research protocols and determining the conditions under which neural implants should be explanted, yet little is understood about how RECs make these decisions. To better understand the role of RECs in explantation decisions of neural implants, we approached REC secretaries within the Netherlands via email, with a list of open-ended questions of which the explantation of neural devices, on informed consent and post-trial care and responsibilities, and psychological harm associated with such trials. The findings highlight the differential technology-specific safety assessments conducted for different types of neural devices. Variability was observed in plans regarding clinical follow-up, post-trial access, and explantation options. While RECs emphasized clear participant information on device maintenance and longevity, the timing of this disclosure varied. Additionally, the psychological impact of explantation was rarely addressed in REC assessments, indicating a gap in ethical oversight. These results shed light on some remaining gaps and suggest the need for improvement in achieving more consistent and comprehensive evaluations of neural device clinical trials, particularly regarding explantation and post-trial access.

**Supplementary Information:**

The online version contains supplementary material available at 10.1007/s12152-025-09619-z.

## Introduction

Neural implants are increasingly considered promising therapeutic interventions for neurological and psychiatric disorders [27]. As clinical trials for neural implants become more common, including devices like deep brain stimulators (DBS), spinal cord stimulators (SCS), and intracortical microelectrodes, concerns about their explantation are growing among patients, researchers, clinicians, manufacturers, and research ethics committees (RECs). After a trial ends, several outcomes are possible: explantation, continued access with ongoing support, or inactivation while keeping the device implanted. Each of these options presents unique ethical and medical challenges.

Explantation involves an invasive procedure and may be recommended by the study investigators or chosen by the participant, due to the ending of a study, medical complications, lack of therapeutic benefit, or personal and psychological reasons [9, 24]. Conversely, continuing use or inactivating the device without removal may avoid surgical risks but raises questions about long-term monitoring, maintenance, and post-trial support [15]. These challenges are closely related to discussions about post-trial access, with scholars raising attention to the ethical challenges of continued access to neurotechnology [11, 15, 20].

Given the potential harm associated with explantation and the ethical considerations surrounding continued use, a comprehensive assessment of trials involving neural implants is urgently needed. Yet, little is known about how RECs assess these post-trial options, particularly regarding explantation. An understanding of this knowledge gap is warranted especially as the need for specific guidelines for RECs to assess these provisions has been previously called for [15].

RECs serve as independent oversight bodies responsible for protecting participants and ensuring the ethical integrity of clinical research based on the medical ethics principles [1]. Their primary functions include reviewing study protocols, assessing and weighing risks and benefits, and ensuring compliance with ethical and regulatory standards. RECs are composed of multidisciplinary members with expertise in both scientific and nonscientific areas—including clinicians, legal experts, ethicists, patient representatives, caregivers—which enables them to integrate diverse perspectives in their deliberations [16]. Despite various challenges such as lack of resources and increased administrative burdens, RECs are still considered a crucial force in regulating research and ensuring the safety of participants. In clinical trials involving neural devices, there is a growing call for RECs to assess not only efficacy and safety but also a broader range of risks. Neural implants in particular require attention to phenomenological changes, algorithmic bias, cybersecurity vulnerabilities, and issues arising from industry-led research, including conflict of interest, liability, and post-trial duties [12, 23].

This study employed an innovative empirical approach by directly engaging with RECs and systematically collecting their responses to targeted questions on the assessment of neural device explantation and other post-trial options. We investigated whether and how RECs in the Netherlands evaluate these considerations before granting approval for a study. The questions we asked the RECs were based on recent discussions in the explantation literature. By investigating written responses to questions about the research protocols, we explored how ethical considerations are assessed, with a focus on explantation, post-trial access and support, psychological harm, and roles and responsibilities of stakeholders of a clinical trial. These responses reflect the rationale behind REC evaluations, and therefore provide valuable insights into REC practices when assessing neural implant studies. With this novel approach, a better understanding of institutional decision-making processes are identified, followed by bridging the gap between theoretical debates and ethics review in practice.

## Methods

### Overview of RECs in the Netherlands

**Fig. 1:**
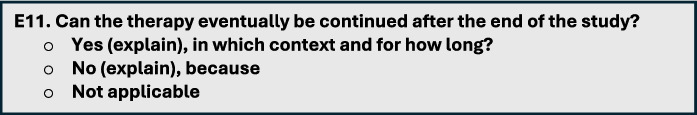
Question (E11) on post-trial access in the ABR form for research ethics committee assessment and registration

In the Netherlands, a total of 14 independently functioning RECs assess medical research protocols and additional forms of studies with human subjects that have been submitted by principal investigators. Although most RECs are affiliated with institutions such as academic medical centers or hospitals, their jurisdiction typically extends beyond their home institution, and in practice they review protocols from both academic and non-academic organizations nationwide. These RECs are comprised of medical experts, lawyers, ethicists, methodologists, a research participant representative and when necessary additional expertise in areas such as pharmacology, psychology and medical devices. Generally, RECs organize one round of written questions that is shared with the researchers and based on which they can still adjust and improve their protocol, a written response of the researchers with their integrated amendments and explanations is requested. The secretary of the REC coordinates this process and ensures all relevant data is safely shared and stored. If the research protocol is not of sufficient quality, deemed unscientific or raises insurmountable ethical concerns, the committee rejects the application, meaning that the research project cannot be conducted. As part of their assessment, provisions regarding explantation and post-trial arrangements are discussed by Dutch RECs in the general assessment and described in the registration form (also known as ABR form) (http://www.ccmo.nl) (See Fig. 1).

### Data Collection

**Fig. 2:**
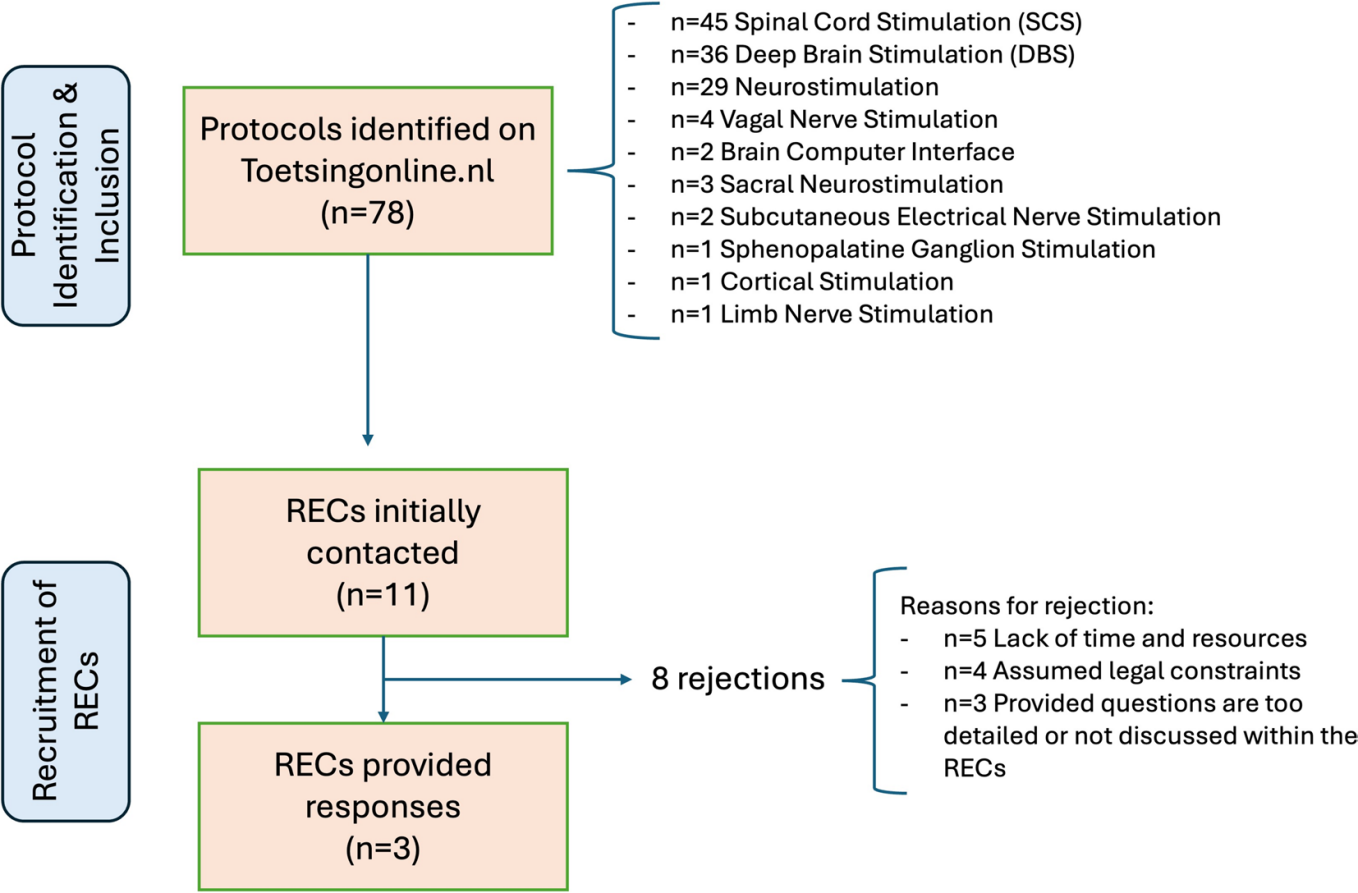
Flowchart of data collection and overview of types of neural implants included in the selected research protocols. RECs Research Ethics Committees

To identify REC secretaries eligible to respond to our questions, we evaluated which RECs have assessed protocols with neural devices in the past by searching the open register of medical research trials (Toetsingonline.nl) in September 2023. Within this portal we searched for protocols using implantable neurotechnologies as search terms including “Brain computer interfaces”; “deep brain stimulation”; “spinal cord stimulation”; and broader terms like “neurostimulation”. Research protocols were screened and selected when the experimental procedure involved the implantation of neural devices. No restrictions were applied regarding type of use (diagnostic or interventional) or study design (prospective or observational); all protocols involving implantation were eligible for inclusion. Our search of the online portal register identified 78 eligible research protocols, with the majority corresponding to SCS trials (n = 31) and the minority to BCI trials (n = 2) (Fig. 2). The name of the corresponding REC was retrieved and the contact information was collected from the respective REC website. Figure 2 provides a visual overview of the data collection process, including the identification of eligible research protocols, REC responses, and the types of neural implants represented in the final sample.

REC secretaries (n = 11) were contacted via email including the numbers of the selected study protocols and questions (Supplementary Table 1). The questions focused on whether the following ethical considerations were discussed and assessed during the evaluation of submitted research protocols: (1) explantation, (2) post-trial access and support, (3) psychological harm, and (4) roles and responsibilities of stakeholders of a clinical trial (Supplementary Table 1). For each protocol, the questions were answered by REC secretaries with information taken from documents provided with the research protocols, internal committee hearings, and the question list provided to the researchers from the committee members.

### Data Analysis

In analyzing the responses provided by RECs on how they assessed research protocols, we conducted a thematic analysis to identify and cluster various reasons mentioned by the RECs. We combined deductive and inductive analysis by drawing on reasons, concerns and implications of explantation and post-trial access and support of neural implants.

## Results

### General Information

Only a 3 RECs agreed to provide responses for the selected protocols, with the rest (n = 8) providing different reasons for not taking part. This resulted in 29 research protocols included in the analysis (a summary can be found in Fig. 2). These studies were funded by both academic and non-academic organizations.

Of the information retrieved from the 3 RECs, it becomes clear that not all topics (explantation, post-trial access, post-trial care and support, psychological harm, roles and responsibilities) were equally discussed across the protocols (Table 1).

**Table 1:** Overview of the number of studies and percentages assessed in RECs discussing ethical concerns relating to implantable neural devices

	Explantation	Post-trial access	Post-trial care and support	Psychological Harm	Roles and Responsibilities
Discussed	8 (27,6%)	12(41,38%)	13(10,34%)	8(27,6%)	18(62,1%)
Not Discussed	20 (69%)	14(48,28%)	15(51,72%)	21(72,41%)	9(31,03%)
Not applicable	1(3,45%)	3(10,34%)	1(3,45%)	0(0%)	2(6,9%)

### Explantation

Our analysis shows that explantation is not often discussed in Dutch RECs. Reasons relating to the explantation of a device may include critical aspects like specific physical and psychological conditions for (not) explanting the device, long-term availability of the device to participants, ongoing clinical support, management of potential complications, and withdrawing or continuing the intervention after the trial ends. Not discussing explanation in the protocol or REC meeting can mean that patients are simply followed up with standard care (n = 20).

When explantation reasons were discussed, RECs required researchers to improve the communication with participants concerning reasons relating to explantation and the potential consequences of discontinuing their participation in the study. Particularly, for DBS studies, RECs requested that patients be informed about how the electrodes will be managed after the end of the trial. When the possibility of explantation was discussed, the response of the researchers was that removal of the implant was possible but switching the DBS off would be more convenient (n = 1; n referring to number of protocols). For both BCI studies, the safe removal of the device was discussed and the possibility for study participants to decide for themselves to continue using the device after the end of the study.

### Continued Access, Care, and Support

Post-trial considerations in implanted neural devices typically refer to device maintenance, continued access to the device, continued support for the patients, and financial considerations. For DBS and SCS devices, post-trial considerations were not discussed in detail among the RECs, specifically because receiving a DBS or SCS device was considered as ‘standard care’ for the condition and even if participants decided to withdraw from the trial, they would be able to receive continued access and support through standard medical care procedures. However, for one of the SCS studies, the question about the financial costs of the treatments to be tested in the study was raised. The REC in this case suggested that information on the differences in costs be provided to the participants, especially if there are differences in costs between both study interventions. Whereas for BCI trials (n = 2), post-trial considerations were always discussed. Some of the points discussed by the REC concerning the BCI trials included ensuring that participants were clearly informed during the consent process that, at the end of the trial, the device would be safely removed. This REC (n = 1) also requested that post-trial access is ensured and provided according to patient’s wishes, and at no cost for them under the guidance of the research team. The REC also requested to confirm that the materials are effective and can remain in place long-term, and investigate whether the costs associated to any damage will be reimbursed.

Moreover, in one case, the committee pushed for post-trial access by objecting the removal of the implant after one year, stating that this conflicted with the principles laid out in the Declaration of Helsinki, especially when participants benefit from the implant, provisions should be made for continued access. However, in another case, paraplegic volunteers agreed to take part in a short-term trial with the aim of investigating physiological functioning without therapeutic benefit, and it was determined by the research team that the electrodes would be removed after five days, at the end of the trial. In another BCI study, it was recommended to provide participants with scenarios and options for them to decide what to do with the device at the end of the trial. These options could include: (1) surgical removal of all implanted components; (2) partial removal, leaving certain parts in place; (3) continued use of the fully implanted device with post-trial maintenance provided as needed; or (4) leaving the device and all its components implanted but inactivated. In consultation with the patient, family, and the neurosurgeon, a decision can be made, preferably under psychological guidance organized and approved by the committee. In this case (BCI trial), participants were given options regarding the post-trial access early on, before the trial began, however this was not the case for all the analyzed trials. In one SCS trial in particular, participants were only informed about their post-trial options after the trial had concluded.

For the BCI studies, the REC ensured that specific arrangement were in place to facilitate post-trial access. This agreement can be extended annually in consultation with the study participant and insurance, and as such will continue as an extension of the study in question. It was also mentioned in the response to a BCI study (n = 1) that post-trial access is still possible in a research context, according to the Medical Devices Regulations (MDR), where participants can still be covered and cared for by the insurance and researchers or clinicians. Moreover, a lack of CE marking (an indication of conformity to European legislative requirements) of a device was considered insufficient to discourage participants from keeping their implant.

RECs refrained from further discussing post-trial considerations when section E11 of the ABR form (Fig. 1) clearly stated that post-trial access was possible and desired by the study participant. However, in certain cases, the committee did require researchers to clarify participants information about the long-term use of the device, including status of the electrodes, battery replacement, and the device’s lifespan. In one particular case, the REC noted inconsistencies in the information about battery life provided to patients, where both a period of 3–6 years and up to12 years were communicated. For one of the BCI studies, when asked about post-trial support, it was reported that post-trial support was not going to be needed.

### Psychological Harm

Psychological harm related to the explantation of neural devices includes emotional distress, identity changes, and anxiety about device removal or loss of function. Responses from RECs regarding psychological harm were concerned with implantation of neural devices rather than explantation. For example, for one DBS study the REC requested that patients receiving DBS should know that they are able to discontinue participation in the trial if they get too tired. Implantation-related concerns were also discussed in relation to the procedure being performed under local anesthesia and the possibility of that being experienced as psychologically stressful, in addition to the psychological burden associated with undergoing an implantation procedure while awake. With regard to psychological harm, the risk for Parkinson’s patients with mild dementia to have cognitive problems after a DBS surgery were also discussed in one case. Interestingly, in one of the committee meetings, a research participant member requested that researchers investigate whether DBS implant can cause any kind of psychological harm to the patient.

For both BCI trials, it was mentioned that the possibility of psychological harm is assessed. Moreover, the REC highlighted that additional measurements taken next to the main procedure can be seen as contributing to increased psychological burden.

### Roles and Responsibilities for Post-Trial Arrangements

Standard care was provided to patients that choose to continue the use of the device, particularly for SCS and DBS trials. For DBS trials in particular, the roles and responsibilities of different stakeholders were either not further discussed because the submitted research protocol provided sufficient information according to the REC or was dependent on the availability of CE marking for the device. For example, if a device had CE marking, study participants would receive standard care post-trial. However, in case a device did not have CE marking, study participants had the choice to continue with the intervention, as long as it remained in the context of a research study. In the event that patients chose to continue the treatment, technical and clinical support had to continue, i.e. post-trial provisions. That being said, according to RECs, CE marking creates a precedent as to whether post-trial provisions are explicitly required prior to the commencement of a clinical trial, and as such the role and responsibility of stakeholders more clearly defined. For some SCS trials, the responsibilities of stakeholders were clearly stated, for instance sponsors are responsible to cover costs of explantation and post-trial support for a certain duration after the trial, which then becomes the responsibility of the insurer, as is standard procedure.

In one research protocol, the REC reported that the manufacturer of the device could terminate the agreement (related to the costs of the system and battery) if the continuation of the intervention is deemed inappropriate, impractical or inadvisable. The REC requested the research team to make use of the conditions that are formulated in the CCMO (Central Committee on Research Involving Human Subjects) model contract that does not allow such termination.

For BCI trials (n = 2), it was mentioned that all stakeholders (incl. researchers, partnering companies, and insurance companies) had been assessed and would remain involved post-trial since the continuation of the use of the device will be in a research context. Specifically, it was mentioned [by researchers] that the frequency of visits and level of support would be reduced in post-trial care as it can be assumed that the patient would benefit from the device. The other study also assessed the different stakeholders and was reported to still be ongoing for post-market surveillance.

## Discussion

Research with implantable neural devices is increasingly being conducted with human participants. These trials are presenting unique ethical challenges, particularly concerning explantation and post-trial access. While this topic has gained more and more attention in the bioethics literature, the role of RECs and their assessments of similar studies was unexplored. This explorative study examined the deliberations of Dutch RECs in their assessment of explantation provisions in research proposals involving implantable neural devices. Although a few RECs provided responses for the selected protocols, the majority declined to participate mainly due to resource scarcity and perceived legal challenges. This highlights the hurdles in obtaining comprehensive insights into how RECs evaluate similar trials, which impedes a comprehensive over view into their decision-making processes, the consistency of their assessments, and the criteria they use to ensure ethical standards are met across different studies. Consistent with previous literature on explantation and post-trial considerations of implantable neural devices, similar themes emerge in our study. For instance, a recent survey by Higgins et al. [11], reports that researchers were required by their REC to include plans concerning post-trial provisions in their research protocol, however this was not the case for non-governmental sponsors [11]. Moreover, researchers have argued that RECs should make it a requirement to include post-trial plans in their submissions [15]. Moreover, the need for increased oversight from RECs for similar high-risk trials that may even involve personality altering effects has been frequently argued for [2, 20, 22].

Our study further strengthens the evidence that post-trial access to neural implants is currently not guaranteed, risking leaving patients post-trial in a state worse than prior to the trial [9]. As has been previously observed, this could be due to a number of reasons, including underdeveloped healthcare infrastructures, lack of finances, and poor collaboration between stakeholders [11]. Clinical trials are generally structured along a network of legal agreements involving multiple parties, including researchers, sponsors, trial sites, and participants. These agreements, which can vary widely (especially across different jurisdictions), primarily define legal rights and obligations of each party, though fundamental principles are outlined in amongst others the Good Clinical Practice Guidelines (GCP, 2001). In Europe for instance, medical devices must comply with the MDR (MDR 2017/745) and obtain a CE mark to demonstrate that they meet the required safety and performance standards. In certain cases CE marking is not required, such as for devices that are still under development (e.g., iBCIs) and being used for clinical research or compassionate use reasons [5]. With respect to legal considerations, post-trial arrangements may require different provisions depending on the device in question, which is why it has been suggested to include clear provisions for explantation and post-trial access and support within plans [2]. Our study indicates that these provisions vary depending on the technology, in terms of whether these are arranged, how detailed these arrangements are, and for which period of time these arrangements are in place.

### Tech-Specific Assessments

Discussions within RECs about explantation considerations, post-trial access and support, psychological harm, and roles and responsibilities of stakeholders were mainly dependent on the type of implant, including whether it had received CE marking or not. For instance, increased discussions on post-trial considerations for BCI devices (and not DBS and SCS) could be due to the absence of regulatory approval for BCI devices for non-research purposes, and hence are not considered as ‘standard care’ procedure. Despite lack of CE marking, RECs mentioned that study participants should still have access to the device if they wish, as long as it is being performed within a research context. This highlights that a need for technology-specific safety assessments is essential given that each technology is paired with its own ethically relevant considerations and risks. This distinction is noteworthy, as concerns relating to explantation and post-trial access in BCI studies seem to be discussed more frequently than in DBS studies. One possible reason for this difference is that DBS is increasingly viewed as standard care procedure, with established safety and clinical guidelines. In contrast, BCI devices are still largely experimental and lack regulatory approval for non-research purposes, leading to heightened scrutiny and more extensive discussions within RECs regarding explantation, post-trial access and support.

### Research Ethics Requirements

For BCI trials, there was no mention of the specific post-trial access provisions that should be established in case a stakeholder terminates the agreement or if patients discontinue or withdrawn from the research trial. There has been increasing reports in the academic literature on the need for post-trial provisions [5], also in the field of neurotechnology [10, 14, 15]. Moreover, informed consent forms in many similar trials currently do not sufficiently elaborate on explantation options and post-trial considerations [10, 11]. Our study underlines that post-trial arrangement vary not only per application and study type, but also in terms of information requested about post-trial responsibilities. Our study also indicates that the plans differ much, in terms of clinical follow-up after withdrawal, post-trial access for those benefitting from the neural implant and possibilities for explantation after the trial. In contrast, the National Institutes of Health (NIH) BRAIN Initiative grant application guidelines outline that researchers need to set up a plan that specifically deals with “ethical and practical considerations of invasive device maintenance and ultimate removal”, including long-term plans for supporting patients during and after the trial [20]. In line with this, the importance of RECs to systematically and consistently inquire about (long-term) post-trial plans has been emphasized in the literature [10, 21], in international guidelines such as the CIOMS guidelines [3], and in recent regulatory discussion, including the UK’s Regulatory Horizons Council on Neurotechnology Regulation[18].

### Respecting Participant Autonomy

Issues with informed consent, particularly concerning implantable brain devices, have been repeatedly discussed in the literature [2, 10, 13, 19, 26]. Issues including wrongful management of expectations, providing insufficient information, and affective impairment can all influence an individual’s autonomy and may question the legitimacy of the consent provided. Our study showed that RECs demanded that study participants are fully informed about post-trial implications, by requesting that the provided documentation (participant information, informed consent, etc.) is clear, and that consistent information about device maintenance and longevity is provided. The acceptable timing of such information however varied, as our study indicated that for one study participants were only informed about post-trial options after ending the trial. This is not always unproblematic, for example when post-trial options is relevant information that participants want to weigh in their decision to participate. Furthermore, it has been previously argued that participants should have influence over the decision to explant or not [8]. For a BCI trial, the REC highlighted the need to provide participants with explantation options, including switching the device off, partial explantation, or a full explantation of all parts of the system. All of these provisions highlight the need for increased attention from RECs to respect study participant’s autonomy in the assessment of post-trial options and pre-trial information provision. That also underlines the need for adequate communication with study participants to ensure that they are properly informed about the research process and the risks involved.

### Psychological Harm

Psychological harm is mainly understood in relation to implantation of neural devices which is not representative of all harms that patients may be experiencing within these trials. Psychological harm related to explantation may not be limited to immediate effects but also arise from the distress or fear of living without the device [7]. Hansson [9] argues that the assessment of risk associated with explantation should not be limited to the immediate effects of the surgery itself. For instance, explantation of breast implants due to the increased risk of rupture should be weighed against the harmful psychological effects of the removal of the implant on the patient’s body image [4, 25]. Having a better understanding of the psychological impact of the explantation of brain devices (DBS, BCI) is of great importance, especially that it can be associated with a negative experience. Our study has shown that in current REC assessment the psychological impact of explantation on an individual’s well-being, autonomy, sense of self is rarely discussed.

It is important to acknowledge that ethical applications for neural device research are diverse, and RECs assess them on an individual basis, taking into account the unique aspects of each study. However, our findings suggest there is room for improvement in achieving more consistent and comprehensive evaluations, particularly regarding explantation and post-trial access. While the results highlight important gaps and concerns, a more comprehensive overview including additional RECs would provide further valuable insights.

### Strengths & Limitations

One limitation of this study is that our analysis includes only research protocols assessed by three Dutch RECs, thereby being unable to provide a complete overview of considerations for the explantation of neural devices across all Dutch RECs. Due to limitations in resources and the interpretation of legal restrictions, many RECs were not able to provide us with the required information about the identified studies. As we did not have access to the original protocols or REC deliberation transcripts and notes, and relied on summaries and responses provided by REC secretaries, we cannot determine in what context the considerations such as explantation and post-trial access were raised. The studies analyzed are representative of the larger sample of eligible studies in terms of the variety of neural devices (i.e., BCI, SCS, VNS), university and non-academic organizations initiated studies, the types of illnesses being investigated (i.e., epilepsy, Parkinson’s disease, locked-in-state). The results of this analysis do highlight important gaps and concerns in relation to explantation and post-trial access, a more comprehensive overview including different RECs would still be of added value. A key strength of this study lies in its innovative method and empirical design: by collecting structured input from RECs on neural device explantation, we capture institutional processes that are often inaccessible in neuroethics research. This approach not only advances methodological diversity in the field but also offers a replicable model for studying other ethically challenging technologies.

## Conclusion

The level of discussion about explantation of neural implants within RECs influences the procedures and conditions for explantation of neural implants and ultimately the associated risks and harms for participants. Many of the concerns emerging from this study point to neural device abandonment—which includes failures in informed consent, and lack of ongoing medical, technical, or financial support during the device’s lifespan [17]. Insights into the assessment of research protocols within RECs is an important step in facilitating the identification of gaps and ethical concerns that might require increased attention. Clinical trials with neural implants should be evaluated while taking into consideration the possibility of explantation, the need for post-trial care and provisions, associated psychological harm, and respect for the participant’s autonomy. By capturing how RECs deliberate on the explantation of neural devices and post-trial considerations, this research provides an empirical foundation that can substantively inform the development of ethical guidelines and frameworks that can be adopted by RECs to assess future studies. Furthermore, our insights can foster establishing standardized protocols for evaluating research protocols with implantable neural devices, to better inform decisions about explantation and post-trial considerations of neural devices, in the Netherlands and abroad. Future avenues should investigate whether imposing requirements by RECs in the early phases of a clinical trial is desirable to ensure ethically sound procedures for the explantation and post-trial care of neural implants.

## Supplementary Information

Below is the link to the electronic supplementary material.

Supplementary file1 (DOCX 15 KB)
