# Supplementary material for: To Explant or not to Explant Neural Implants: an Empirical Study into Deliberations of Dutch Research Ethics Committees
Source: Neuroethics. 2025 Oct 10;18(3):45. doi: 10.1007/s12152-025-09619-z (PMC12513904; doi:10.1007/s12152-025-09619-z)
Supplement: Supplementary file 1 — Supplementary file1 (DOCX 15 KB) [file 12152_2025_9619_MOESM1_ESM.docx]

Table 1. Themes Discussed by Research Ethics Committees in Relation to Neural Device Protocols

| Requirements and considerations regarding post-trial care and support (including costs) | Decisions regarding explantation of the device (e.g. will the device be explanted after the end of the trial and why/why not) | Weighing and considering the wishes of research participants regarding post-trial use of the device | Assessment of psychological burden of the study (e.g. anxiety and stress, loss of agency, etc.) | The role and responsibilities of those involved with regard to post-trial care (including researchers, involved companies and health insurers) |
| --- | --- | --- | --- | --- |
